# Mechanism of *Tripterygium wilfordii Hook.F.- Trichosanthes kirilowii Maxim* decoction in treatment of diabetic kidney disease based on network pharmacology and molecular docking

**DOI:** 10.3389/fphar.2022.940773

**Published:** 2022-10-28

**Authors:** Lingfei Lu, Jinting Peng, Peijun Wan, Hongcheng Peng, Jiandong Lu, Guoliang Xiong

**Affiliations:** ^1^ Department of Nephrology, Shenzhen Traditional Chinese Medicine Hospital, Guangzhou University of Chinese Medicine, Shenzhen, Guangdong, China; ^2^ The Fourth Clinical Medical College of Guangzhou University of Chinese Medicine, Shenzhen, Guangdong, China; ^3^ Department of Gynecology, Shenzhen Traditional Chinese Medicine Hospital, Guangzhou University of Chinese Medicine, Shenzhen, Guangdong, China; ^4^ Department of Nephrology, Shenzhen Traditional Chinese Medicine Hospital, Affiliated to Nanjing University of Chinese Medicine, Shenzhen, Guangdong, China

**Keywords:** *Tripterygium wilfordii Hook.F.*, *Trichosanthes kirilowii Maxim*, diabetic kidney disease, network pharmacology, molecular docking, mechanism

## Abstract

**Background:** Diabetic kidney disease (DKD) is the most common cause of end-stage renal disease. The effective treatment of DKD would rely on the incorporation of a multi-disciplinary. Studies have shown that *Tripterygium wilfordii Hook.F.* and *Trichosanthes kirilowii Maxim* have remarkable curative effects in treating DKD, but their combination mechanism has not been fully elucidated.

**Methods:** We explored the mechanism of *Tripterygium wilfordii Hook.F.-Trichosanthes kirilowii Maxim* decoction (Leigongteng-Tianhuafen Decoction,LTD) in the treatment of DKD by network pharmacology and molecular docking. The main active components and action targets of LTD were collected from Traditional Chinese Medicine Systems Pharmacology Database and Analysis Platform (TCMSP) database. The speculative targets of DKD were obtained from GeneCards, DisGeNET, and Online Mendelian Inheritance in Man (OMIM) databases. Then, an herb-component-target network was constructed based on the above analyses. The biological function of targets was subsequently investigated, and a protein-protein interaction (PPI) network was constructed to identify hub targets of DKD. The gene ontology (GO) function enrichment analysis and kyoto encyclopedia of genes and genomes (KEGG) pathway enrichment analysis were performed by RStudio. Finally, molecular docking was performed by AutoDock Vina and PyMOL software to explore the interaction between compounds and targets. Furthermore, the DKD model of human renal tubular cells (HK-2) induced by high glucose (HG) was selected, and the predicted results were verified by western blot analysis and immunofluorescence.

**Results**: A total of 31 active components of LTD were screened out, and 196 targets were identified based on the TCMSP database. A total of 3,481 DKD related targets were obtained based on GeneCards, DisGeNET, and OMIM databases. GO function enrichment analysis included 2,143, 50, and 167 GO terms for biological processes (BPs), cellular composition (CCs), and molecular functions (MFs), respectively. The top 10 enrichment items of BP annotations included response to lipopolysaccharide, response to molecule of bacterial origin, response to extracellular stimulus, etc. CC was mainly enriched in membrane raft, membrane microdomain, plasma membrane raft, etc. The MF of LTD analysis on DKD was predominately involved in nuclear receptor activity, ligand-activated transcription factor activity, RNA polymerase II-specific DNA-binding transcription factor binding, etc. The involvement signaling pathway of LTD in the treatment of DKD included AGE-RAGE signaling pathway in diabetic complications, IL-17 signaling pathway, insulin resistance, TNF signaling pathway, etc. Molecular docking results showed that kaempferol, triptolide, nobiletin, and schottenol had a strong binding ability to PTGS2 and RELA. Furthermore, the *in vitro* experiments confirmed that LTD effectively decreased the expression of PTGS2, NF-κB, JNK, and AKT in the HG-induced DKD model.

**Conclusion:** The findings of this study revealed that the therapeutic efficacy of LTD on DKD might be achieved by decreasing the expression of PTGS2, NF-κB, JNK, and AKT, which might improve insulin resistance, inflammation, and oxidative stress. These findings can provide ideas and supply potential therapeutic targets for DKD.

## Introduction

Diabetic kidney disease (DKD), which accounts for about 40% of diabetic patients, is the main cause of chronic kidney disease (CKD) worldwide. The natural history of DKD includes glomerular hyperfiltration, progressive proteinuria, decreased glomerular filtration rate, and eventually end-stage renal disease (ESRD) (Alicic et al., 2017). DKD is one of the main causes of ESRD and is associated with the high mortality of diabetic patients (Deng et al., 2021). Studies have shown that by 2040, the global prevalence of diabetes is expected to increase to 642 million, of which 30–40% will develop into DKD (Tang and Yiu, 2020). If effective intervention is not given to people with diabetes and DKD, the peak of ESRD caused by DKD may come in the next 10–20 years, which will bring an unbearable burden to the medical and health system. Therefore, it is necessary to explore and develop effective methods to prevent and treat DKD.

In recent years, Traditional Chinese Medicine (TCM) has shown remarkable advantages in treating DKD. Studies have shown that *Tripterygium wilfordii Hook.F.* and *Trichosanthes kirilowii Maxim* have remarkable curative effects in treating DKD. Experiments *in vitro* and *in vivo* have shown that *Tripterygium wilfordii Hook.F.* has powerful immunosuppressive, anti-inflammatory, and anti-proteinuria effects (Lengnan et al., 2020). *Trichosanthes kirilowii Maxim* has hypoglycemic and antioxidation effects (Chen et al., 2014; Sang et al., 2019). The combination of *Tripterygium wilfordii Hook.F.* and *Trichosanthes kirilowii Maxim* has been widely used in the treatment of DKD in TCM, but their combination mechanism has not been fully elucidated.

Network pharmacology is a new method that combines computer science with medicine, constructs, and visualizes the interaction network of multi-gene, multi-target, and multi-pathway to evaluate the molecular mechanism of herbs (Yuan et al., 2017). Because of its complex matrix properties, this method is very suitable for the study of multi-component herbs such as TCM(Boezio et al., 2017). Molecular docking refers to the process of a small molecule docking with a large molecule in space and obtaining the complete value at the binding site, which is used in structure-based herb design (Saikia and Bordoloi, 2019).

In this paper, we initially explore the mechanism of action of *Tripterygium wilfordii Hook.F.-Trichosanthes kirilowii Maxim decoction* (Leigongteng-Tianhuafen Decoction,LTD) for the treatment of DKD by network pharmacology and molecular docking. The main active components and targets of LTD in the treatment of DKD were screened based on network pharmacology and molecular docking, and the pharmacodynamic material basis of LTD in the treatment of DKD was preliminarily verified, to provide ideas for further mechanism research and clinical promotion.

## Materials and methods

### Collection of components and targets of LTD and construction of herb-component-target network

Traditional Chinese Medicine System Pharmacology Database and Analysis Platform (TCMSP) (https://tcmsp-e.com/) was used to extract the active components of LTD the compounds were screened according to the two ADME attribute values of oral bioavailability (OB)≥30% and herb-likeness property (DL)≥0.18. Consulting the literature and searching the TCMSP database, there are few active compounds of *Trichosanthes kirilowii Maxim*. Then the active compounds of *Trichosanthes kirilowii Maxim* were transformed into Canonical SMILES format by PubChem database (https://pubchem.ncbi.nlm.nih.gov/). SMILES was imported into SwissTargetPrediction (http://www.swisstargetprediction.ch/) platform, and the attribute was set as *Homo Sapiens* to predict the potential targets of active compounds and supplement the known targets of unpredicted active compounds according to literature reports (Gfeller et al., 2014) and used the Uniprot database (https://www.uniprot.org/) to corrected the gene names of targets. The target proteins were introduced into Cytoscape3.9.0 software to construct an herb-component-target network and to be analyzed.

### Screening of DKD related targets

In the GeneCards databases (https://www.genecards.org/), DisGeNET databases (https://www.disgenet.org), and OMIM databases (http://www.omim.org), “diabetic kidney disease” and “diabetic nephropathy” was used as the search term to collect disease targets related to DKD. Finally, duplicates from the three databases were removed to obtain the final DKD related targets.

### PPI network construction

The intersecting targets and Venn diagram of LTD and DKD targets were obtained through the online Venn map platform. Intersection targets were extracted and submitted to STRING version 11.5 database (https://cn.string-db.org/) to build a Protein-Protein Interaction (PPI) network. The biological species was set as “*Homo sapiens*”, the minimum interaction threshold was set as “highest confidence” (>0.9), and the isolated proteins were hidden. Then, the PPI network was obtained by Cytoscape3.9.0 software, and the core targets were screened by CytoNCA. The core targets were obtained with Degree, Betweenness, and Closeness greater than or equal to the median as screening conditions.

### GO function enrichment analysis and KEGG pathway enrichment analysis

To further understand the functions of the intersection targets for the treatment of DKD and their roles in signaling pathways selected above, GO function enrichment analysis and KEGG pathway enrichment analysis was performed using RStudio. The biological processes (BPs), molecular functions (MFs), and cellular compositions (CCs) were included in the GO function enrichment analysis. Furthermore, the condition was limited to *p* < 0.01 and *Homo Sapiens*. The data was sequenced according to the number of enriched targets, and the first 20 entries of enrichment pathways were selected to visualize.

### Construction of the herb-compound-target-pathway-disease network and molecular docking

The components of LTD, LTD-DKD-KEGG pathway common targets and pathway were imported into Cytoscape3.9.0 software to obtain the network diagram of herb-compound-target-pathway-disease network. Then, the targets and components in the top 4 of the Degree value were selected for molecular docking, and the 3D structure was downloaded from PubChem and converted to the component structure through PDB format. The receptor file of the core protein would be imported into PyMOL software to remove small-molecule ligands and water molecules, and imported into AutoDockTools software to add hydrogen, then output as a PDBQT file. The molecular docking was conducted by importing into AutoDock Vina software to obtain the minimum binding free energy of the target protein and the ligand. The optimal binding pattern of each protein was plotted by using Discovery Studio 2019 software.

#### Cell culture

Human kidney proximal tubular epithelial cells (HK-2) were maintained with DMEM/F12 medium containing 10% FBS in the conditions: 95% air and 5% CO2 at 37°C in an incubator, and passaged or preserved every 2–3 days. Cells were observed under microscopy and digested with 0.25% trypsin solution for passaging when they reached 80%–90% confluence. HK-2 cells were separated into three groups: normal group (DMEM/F12 medium supplemented with 5.5 mmol/L glucose), high glucose (HG) group (DMEM/F12 medium supplemented with 30 mmol/L glucose), and high glucose (HG)+LTD group (DMEM/F12 medium supplemented with 30 mmol/L glucose, 50 μg/ml LTD).

#### Western blotting

After inventions, the whole cell lysate of HK-2 was obtained using RIPA buffer (Cell Signaling Technology, Massachusetts, USA) and cOmplete™ Protease Inhibitor Cocktail tablets (Roche Diagnostics, Mannheim, Germany). The protein concentration was quantified by the Quick Start™ Bradford Protein Assay Kit (BioRad, California, USA). Then the samples were separated electrophoresed through 10% SDS-polyacrylamide gels, and then transferred to the PVDF membrane (Millipore, Massachusetts, USA). Following blocking in 5% non-fat milk for 1 h at room temperature, and incubated overnight at 4°C with primary antibodies. The primary antibodies included PTGS2 Monoclonal antibody (1:1,000, Proteintech), NF-κB p65 Polyclonal antibody (1:1,000, Proteintech), Phospho-NF-κB p65 (1:1,000, Cell Signaling Technology), AKT Monoclonal antibody (1:1,000, Proteintech), Phospho-AKT Monoclonal antibody (1:1,000, Proteintech), Recombinant Anti-JNK1+JNK2+JNK3(1:1,000, Abcam), Phospho-JNK (1:1,000, Cell Signaling Technology), GAPDH (1:1,000, Proteintech). Then, the membranes were incubated with secondary antibodies (anti-mouse/rabbit IgG, Proteintech) for 1 h at room temperature.

#### Immunofluorescence

HK-2 cells were immunofluorescently stained for phospho-p65 using anti-phospho-NF-κB p65 antibody in a humid environment at 4°C overnight. After being immunostained, the cells were treated for 1 h at 37°C with a secondary antibody that was TRITC (red fluorescence) or FITC (green fluorescence)-labeled. DAPI solution (1.0 g/ml) was then used to stain the cell nuclei for 10 min. A fluorescence microscope (Zeiss, Jena, Germany) was used to capture images of immunofluorescence staining.

## Results

### Component and targets of LTD

The active components of LTD were screened through the TCMSP database. With OB ≥ 30% and DL ≥ 0.18 as the screening conditions, a total of 53 active components were obtained, including 51 from *Tripterygium wilfordii Hook.F*. and 2 from *Trichosanthes kirilowii Maxim*. There were 22 active components without corresponding targets in the database, and 31 active components were finally obtained, as shown in Table 1. The targets corresponding to the active components of LTD were selected by using the UniProt database. Meanwhile, potential targets were added according to the literature, and 151 targets were obtained after the repetitive items were deleted. The potential targets of the active components of *Trichosanthes kirilowii Maxim* were predicted based on the SwissTargetPrediction platform, and the targets and repetitions with a probability of 0 were deleted, and a total of 61 potential targets were obtained. After the repetitive items were deleted, a total of 196 potential targets for the active components in LTD were finally obtained.

**TABLE 1:** Key active components of LTD.

TCM	Mol id	Mark	Main active components	OB%	DL
LTD	MOL000296	LGT1	hederagenin	36.91	0.75
LTD	MOL003182	LGT2	(+)-Medioresinol di-O-beta-d-glucopyranoside_qt	60.69	0.62
LTD	MOL003184	LGT3	81,827–74–9	45.42	0.53
LTD	MOL003185	LGT4	(1R,4aR,10aS)-5-hydroxy-1-(hydroxymethyl)-7-isopropyl-8-methoxy-1,4a-dimethyl-4,9,10,10a-tetrahydro-3H-phenanthren-2-one	48.84	0.38
LTD	MOL003187	LGT5	triptolide	51.29	0.68
LTD	MOL003196	LGT6	Tryptophenolide	48.50	0.44
LTD	MOL003199	LGT7	5,8-Dihydroxy-7-(4-hydroxy-5-methyl-coumarin-3)-coumarin	61.85	0.54
LTD	MOL003208	LGT8	Celafurine	72.94	0.44
LTD	MOL003209	LGT9	Celallocinnine	83.47	0.59
LTD	MOL003217	LGT10	Isoxanthohumol	56.81	0.39
LTD	MOL003224	LGT11	Tripdiotolnide	56.40	0.67
LTD	MOL003225	LGT12	Hypodiolide A	76.13	0.49
LTD	MOL003229	LGT13	Triptinin B	34.73	0.32
LTD	MOL003231	LGT14	Triptoditerpenic acid B	40.02	0.36
LTD	MOL003245	LGT15	Triptonoditerpenic acid	42.56	0.39
LTD	MOL003248	LGT16	Triptonoterpene	48.57	0.28
LTD	MOL003266	LGT17	21-Hydroxy-30-norhopan-22-one	34.11	0.77
LTD	MOL003280	LGT18	TRIPTONOLIDE	49.51	0.49
LTD	MOL000358	LGT19	beta-sitosterol	36.91	0.75
LTD	MOL000211	LGT20	Mairin	55.38	0.78
LTD	MOL000422	LGT21	kaempferol	41.88	0.24
LTD	MOL000449	LGT22	Stigmasterol	43.83	0.76
LTD	MOL002058	LGT23	40,957–99–1	57.20	0.62
LTD	MOL003283	LGT24	(2R,3R,4S)-4-(4-hydroxy-3-methoxy-phenyl)-7-methoxy-2,3-dimethylol-tetralin-6-ol	66.51	0.39
LTD	MOL004443	LGT25	Zhebeiresinol	58.72	0.19
LTD	MOL005828	LGT26	nobiletin	61.67	0.52
LTD	MOL007415	LGT27	[(2S)-2-[[(2S)-2-(benzoylamino)-3-phenylpropanoyl]amino]-3-phenylpropyl] acetate	58.02	0.52
LTD	MOL007535	LGT28	(5S,8S,9S,10R,13R,14S,17R)-17-[(1R,4R)-4-ethyl-1,5-dimethylhexyl]-10,13-dimethyl-2,4,5,7,8,9,11,12,14,15,16,17-dodecahydro-1H-cyclopenta [a]phenanthrene-3,6-dione	33.12	0.79
LTD	MOL009386	LGT29	3,3′-bis-(3,4-dihydro-4-hydroxy-6-methoxy)-2H-1-benzopyran	52.11	0.54
LTD	MOL004355	THF1	Spinasterol	42.98	0.76
LTD	MOL006756	THF2	Schottenol	37.42	0.75

### Construction of herb-component-target network

**FIGURE 1:**
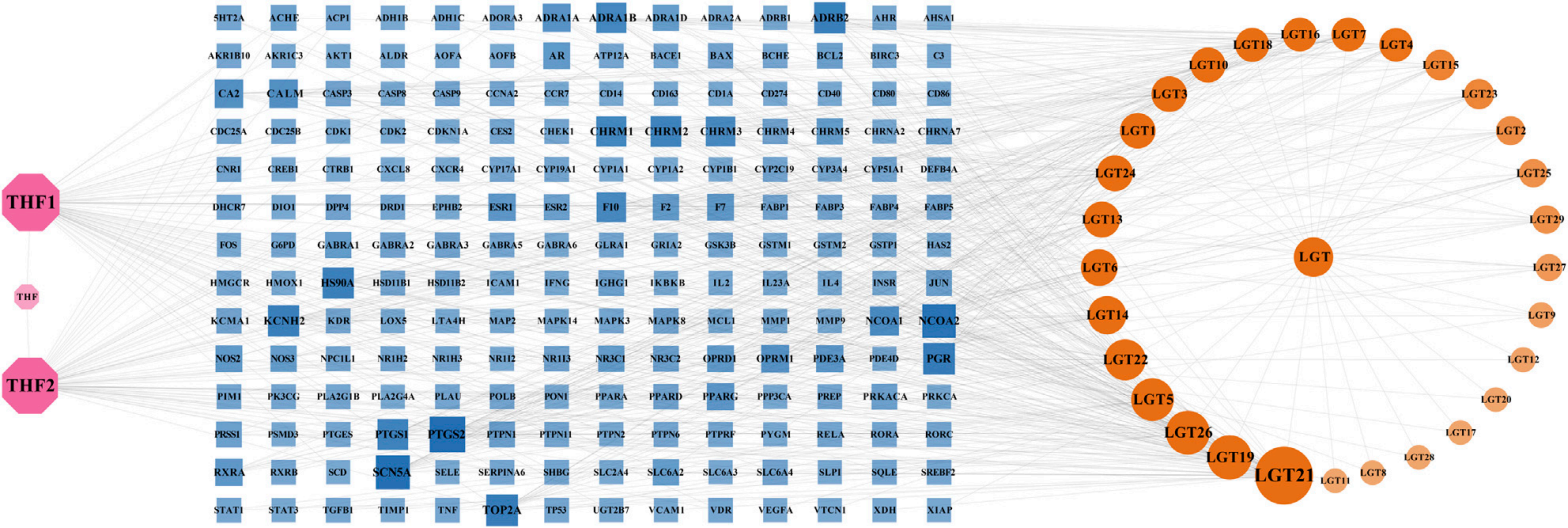
Network of herb-component-target. The pink nodes represented 2 active components of *Trichosanthes kirilowii* *Maxim*, the yellow nodes represented 29 active components of *Tripterygium wilfordii Hook.F*., and the blue nodes represented 196 targets related to the components of LTD. The nodes with a larger area and darker color have a larger degree value.

The obtained active components and potential targets were imported into Cytoscape3.9.0 software, and the obtained herb-component-target network was shown in Figure 1, which contained 229 nodes with 639 edges. The pink nodes represented 2 active components of *Trichosanthes kirilowii Maxim*, the yellow nodes represented 29 active components of *Tripterygium wilfordii Hook.F.*, and the blue nodes represented 196 targets related to the components of LTD. The nodes with a larger area and darker color have a larger degree value. According to herb-component-target network analysis, the active components in the top 6 of the Degree values were kaempferol, spinaterol, schottenol, β-sitosterol, nobiletin, and triptolide, respectively. The top 6 targets of Degree value were prostaglandin-endoperoxide synthase 2(PTGS2), sodium voltage-gated channel alpha subunit 5(SCN5A), nuclear receptor coactivator 2(NCOA2), progesterone receptor (PGR), DNA topoisomerase II alpha (TOP2A), and potassium voltage-gated channel subfamily H member 2(KCNH2), respectively. It was speculated that they might be the important components and possible active targets of LTD.

### Screening of DKD related targets

The GeneCards database obtained 3,422 diabetic nephropathy targets and 13,244 diabetic kidney disease targets. The targets with a relevance score greater than the median were set as the potential targets of DKD, and the final targets were obtained through multiple median screening. After DisGeNET, OMIM, and the database were combined to supplement the related targets, and the duplicate values were deleted after the combination, a total of 3,481 DKD related targets were obtained.

### Construction of PPI network

**FIGURE 2:**
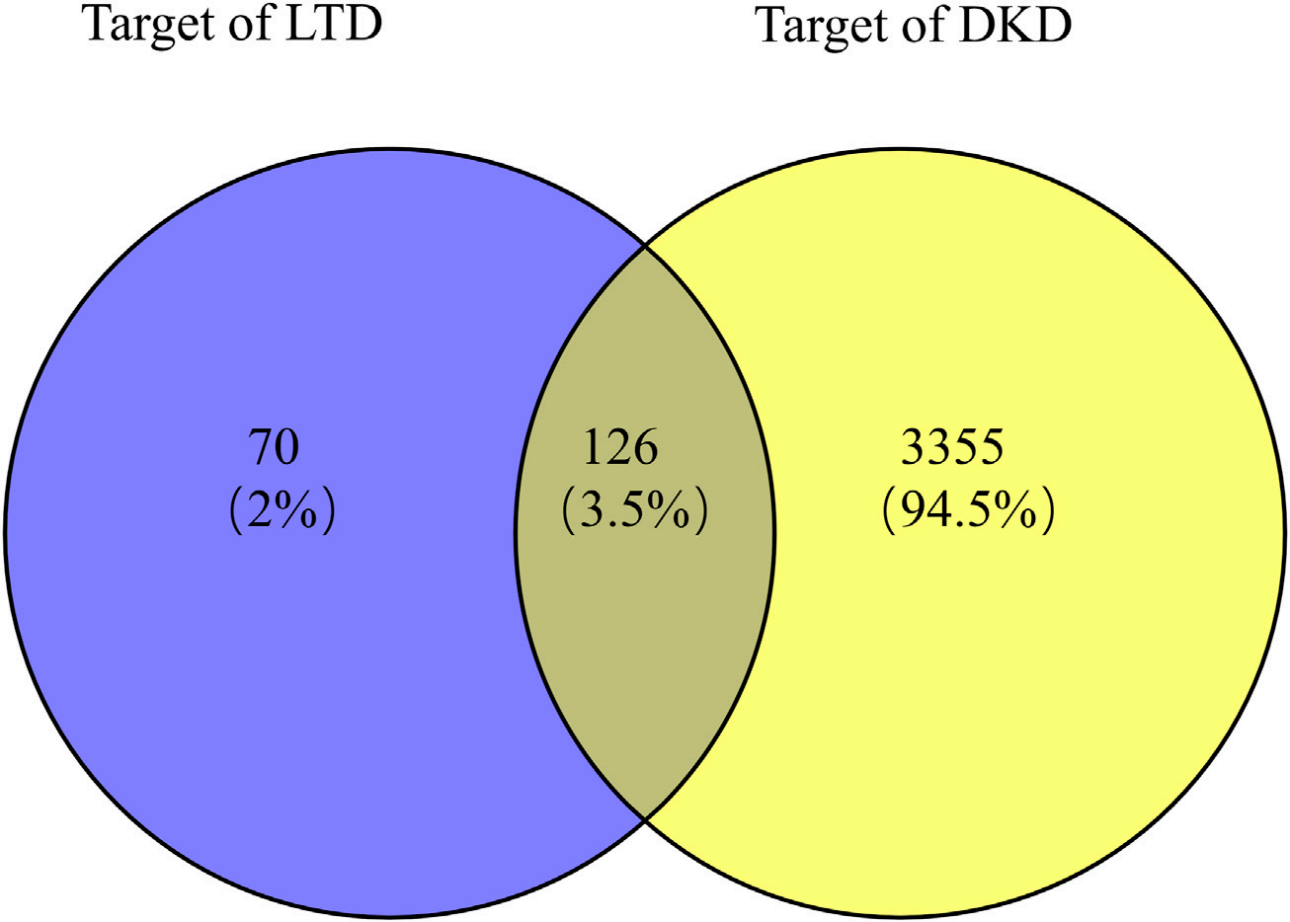
Venn diagram of the intersection target of LTD and DKD.

**FIGURE 3:**
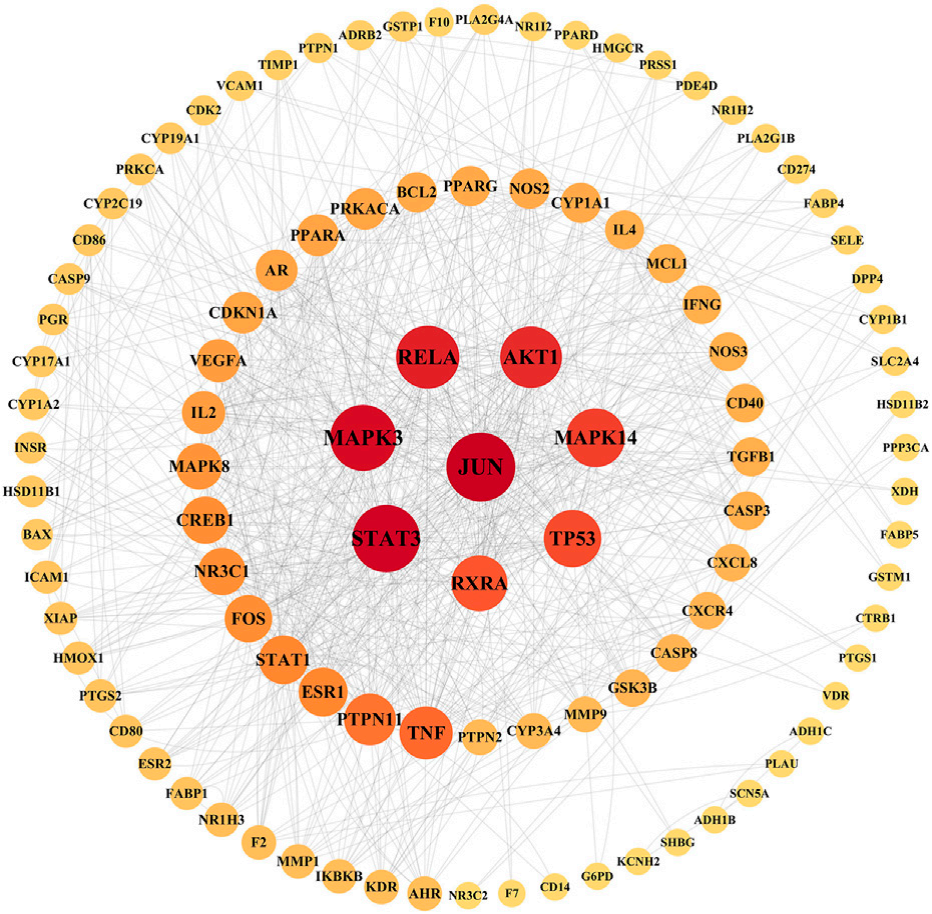
PPI network diagram of target proteins. The nodes with a larger area and darker color have a larger degree value.

Taking the intersection of the LTD and DKD related targets, a total of 126 potential action targets of LTD in the treatment of DKD were obtained, as shown in Figure 2. The 126 potential targets were input into the STRING11.5 database to obtain the PPI network. The PPI network was constructed by using Cytoscape3.9.0 software, as shown in Figure 3. The PPI network consisted of 103 nodes and 738 edges. The CytoNCA was used to screen the core targets, and 39 core targets were obtained with the screening condition of Degree, Betweenness, and Closeness being greater than or equal to the median, which might be the core targets of LTD.

### GO function enrichment analysis and KEGG pathway enrichment analysis

**FIGURE 4:**
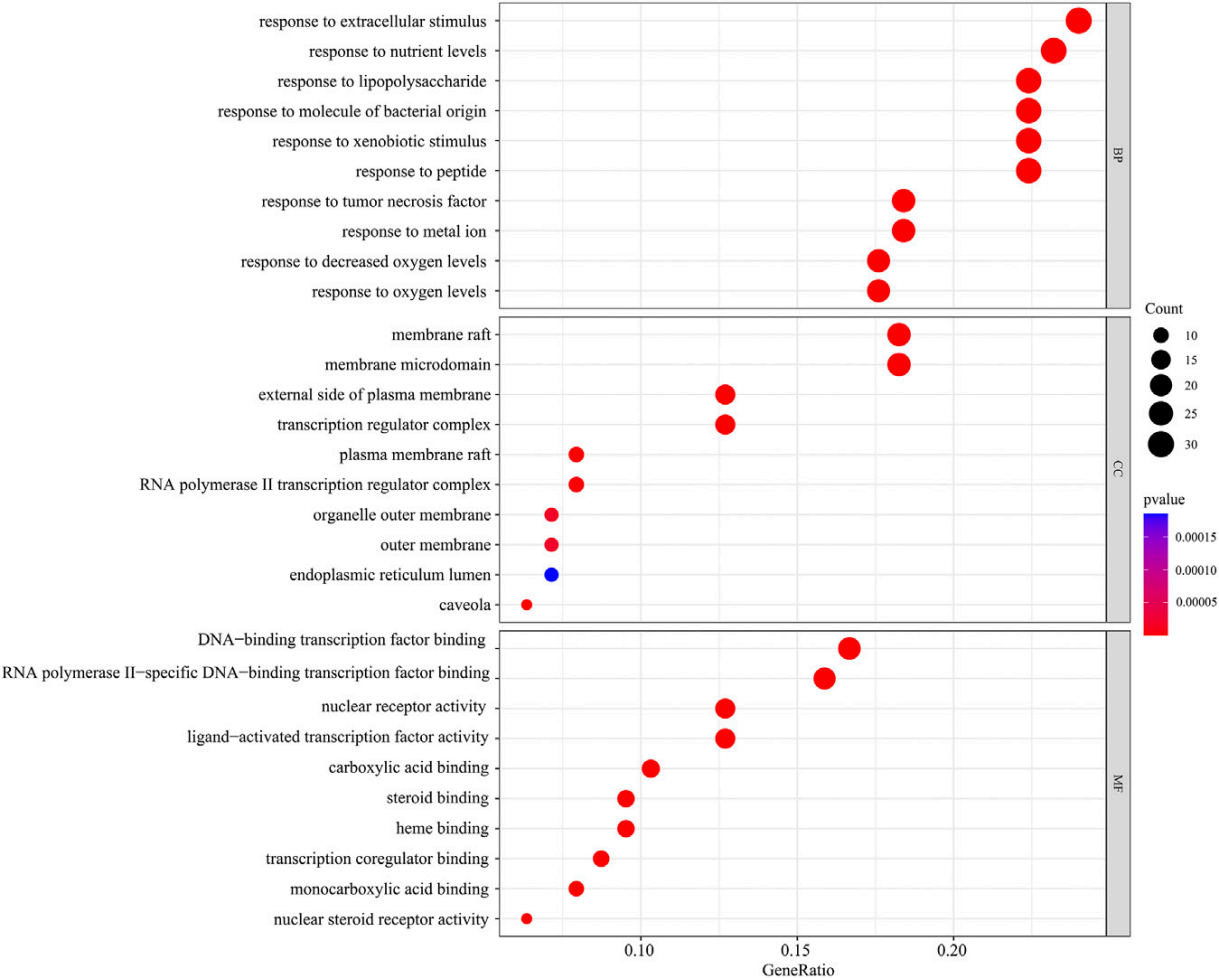
The top 10 pathways for the GO function enrichment analysis based on the common targets of LTD and DKD.

**FIGURE 5:**
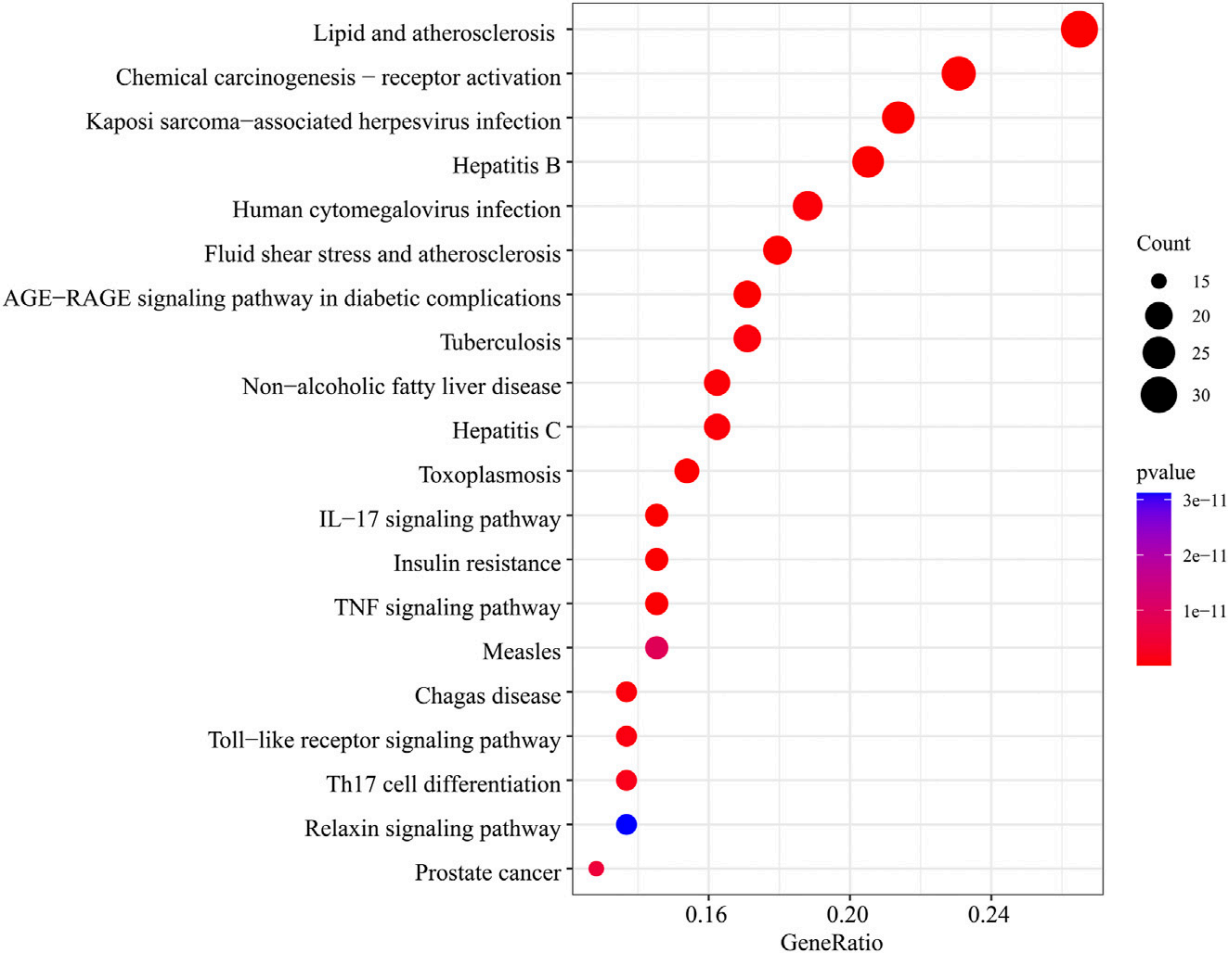
The top 20 pathways for the KEGG pathway are enriched from the common targets between LTD and DKD.

In the GO function enrichment analysis of 126 intersection targets using RStudio, 2,143 BPs, 50 CCs, and 167 MFs were obtained as the target genes. The first 10 entries of the enrichment pathway were selected to visualize the data, as shown in Figure 4. The BPs were mainly related to response to lipopolysaccharide, response to molecule of bacterial origin, response to extracellular stimulus, response to nutrient levels, response to xenobiotic stimulus, response to tumor necrosis factor, response to peptide, response to decreased oxygen levels, response to metal ion, response to oxygen levels, etc. The CCs were mainly related to membrane raft, membrane microdomain, plasma membrane raft, external side of plasma membrane, caveola, transcription regulator complex, RNA polymerase II transcription regulator complex, organelle outer membrane, outer membrane, endoplasmic reticulum lumen, etc. The MFs were mainly related to nuclear receptor activity, ligand-activated transcription factor activity, RNA polymerase II-specific DNA-binding transcription factor binding, nuclear steroid receptor activity, steroid binding, DNA-binding transcription factor binding, carboxylic acid binding, transcription coregulator binding, monocarboxylic acid binding, heme binding, etc. The involvement signaling pathway of LTD in the treatment of DKD included AGE-RAGE signaling pathway in diabetic complications, IL-17 signaling pathway, insulin resistance, TNF signaling pathway, etc. The top 20 pathways were visualized by using bubble charts, as shown in Figure 5.

### Construction of herb-component-target-pathway network

**FIGURE 6:**
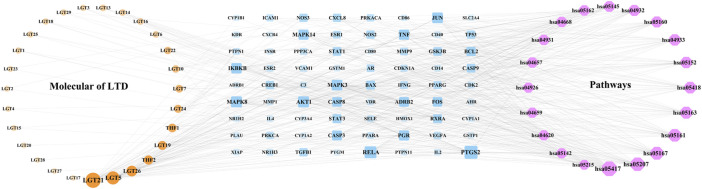
Network of herb-component-target-pathway. The yellow nodes represent the herb component, the blue nodes represent the LTD-DKD-KEGG pathway common target, and the red nodes represents the KEGG pathway. The nodes with a larger area and darker color have a larger degree value.

The components of LTD, LTD-DKD-KEGG pathway common targets and KEGG pathway were imported into Cytoscape3.9.0 software for intersection, and the herb-component-target-pathway network was obtained, as shown in Figure 6. The herb-component-target-pathway network consisted of 87 nodes and 377 edges. According to the analysis of the herb-component-target-pathway network, the active components in the top 4 of Degree values were kaempferol, triptolide, nobiletin, and schottenol, and the top 4 targets for Degree values were prostaglandin-endoperoxide synthase 2(PTGS2), transcription factor p65 (RELA), AKT serine/threonine kinase 1 (AKT1), and mitogen-activated protein kinase 8 (MAPK8).

### Molecular docking

**FIGURE 7:**
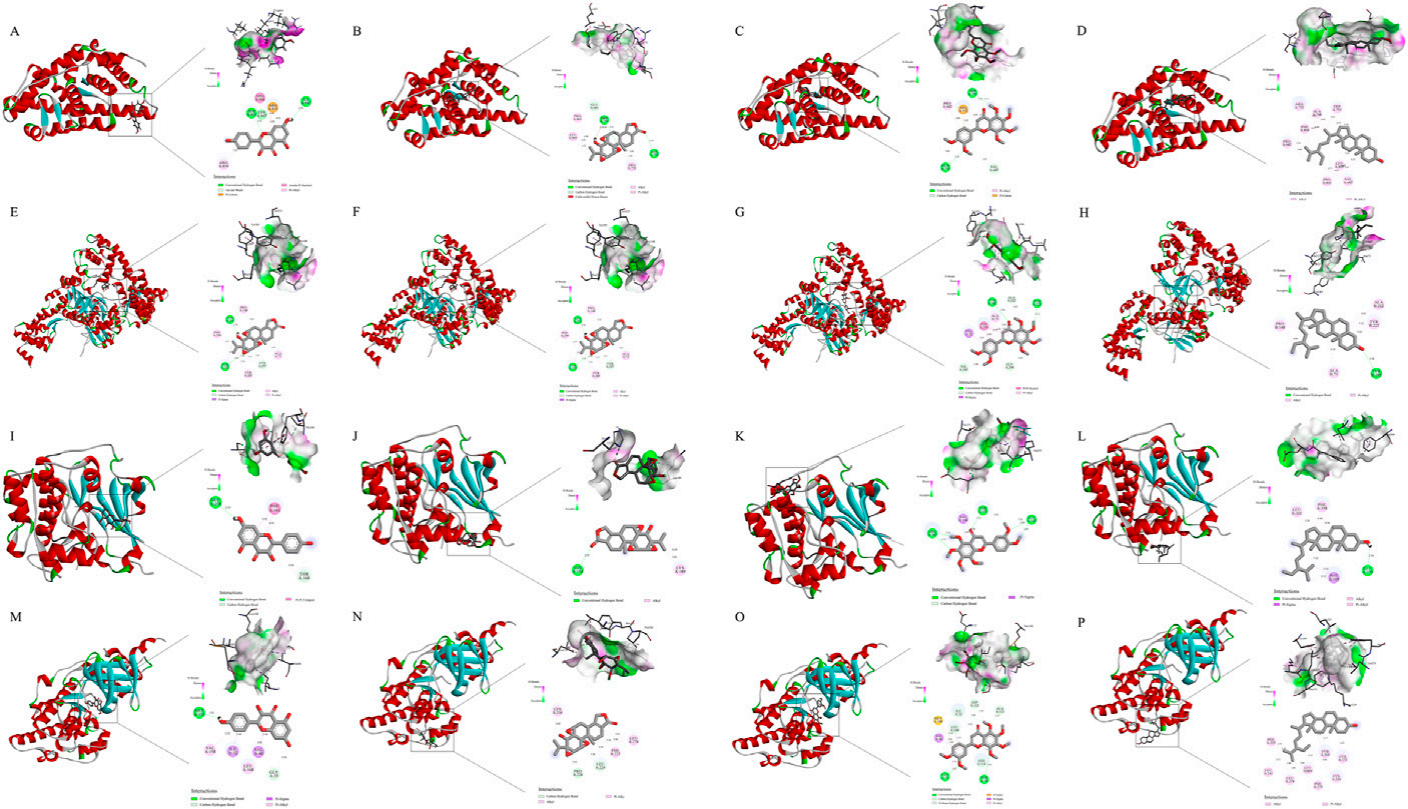
Molecular models of main compounds binding to their predicted key protein targets. **(A)**: molecular docking diagram of PTGS2(5F19) and kaempferol; **(B)** molecular docking diagram of PTGS2(5F19) and triptolide; **(C)** molecular docking diagram of PTGS2(5F19) and nobiletin; **(D)** molecular docking diagram of PTGS2(5F19) and schottenol; **(E)** molecular docking diagram of RELA (3QXY) and kaempferol; **(F)** molecular docking diagram of RELA (3QXY) and triptolide; **(G)** molecular docking diagram of RELA (3QXY) and nobiletin; **(H)** molecular docking diagram of RELA (3QXY) and schottenol; **(I)** molecular docking diagram of AKT1(3CQU) and kaempferol; **(J)** molecular docking diagram of AKT1(3CQU) and triptolide; **(K)** molecular docking diagram of AKT1(3CQU) and nobiletin; **(L)**: molecular docking diagram of AKT1(3CQU) and schottenol; **(M)**: molecular docking diagram of MAPK8(2XRW) and kaempferol; **(N)**: molecular docking diagram of MAPK8(2XRW) and triptolide; **(O)**: molecular docking diagram of MAPK8(2XRW) and nobiletin; **(P)**: molecular docking diagram of MAPK8(2XRW) and schottenol).

Molecular docking was conducted on the key targets and active ingredients in the herb-component-target-pathway network. The protein structures of the core targets PTGS2(5F19), RELA (3QXY), AKT1(3CQU), and MAPK8(2XRW) were downloaded from the PDB database according to the set selection conditions. It is generally believed that the closeness of association between protein and ligand can be represented by the binding energy of docking binding. Generally, lower binding energies will lead to more stable binding conformations and more likely interaction (Yuan et al., 2021). Molecular docking results showed that the docking binding energy of the core compounds of LTD and potential action targets were mostly ≤ -5.0 kcal mol^−1^, which indicated that LTD had a strong binding activity with the predicted action targets. The docking binding energy was shown in Table 2. The molecular docking binding energy showed that PTGS2 and RELA had good binding with kaempferol, triptolide, nobiletin, and schottenol. The molecular docking diagram showing good binding of the main components and main targets in the LTD. was selected as a schematic diagram, as shown in Figure 7.

**TABLE 2:** Docking binding energy of main compounds and key targets of LTD.

Compound	Binding energy (kcal mol^−1^)			
	PTGS2(5F19)	RELA (3QXY)	AKT1(3CQU)	MAPK8(2XRW)
Kaempferol	−9	−9.3	−6.8	−7.9
Triptolide	−9.2	−9.1	−6.7	−7.7
Nobiletin	−8.5	−8.8	−6.2	−7.0
Schottenol	−9.5	−8.4	−7.3	−7.4

### LTD significantly reduced PTGS2, NF-κB, JNK, and AKT expressions in HK-2 cells under HG ambience

**FIGURE 8:**
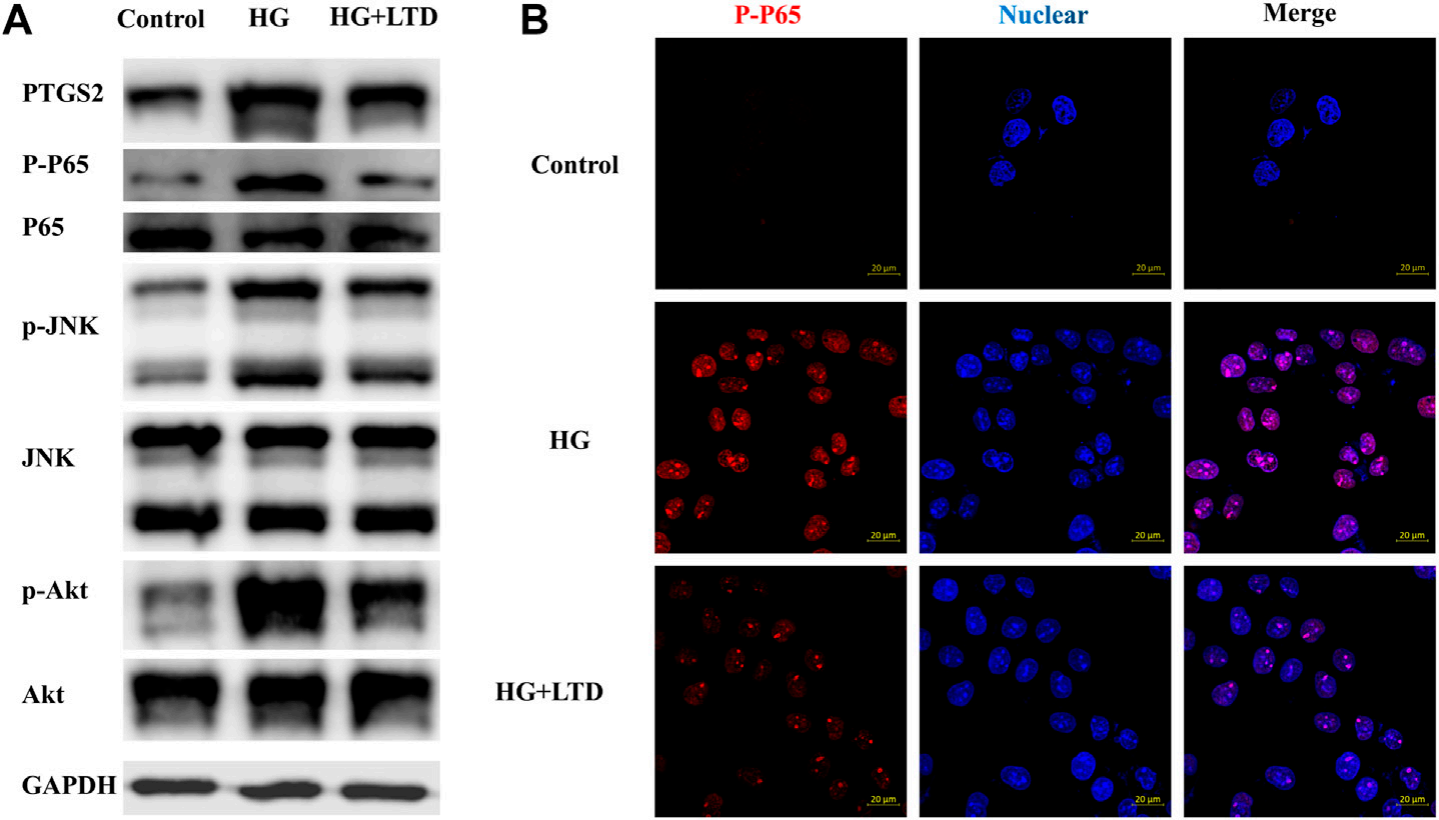
LTD significantly reduced PTGS2, NF-κB, JNK, and AKT expressions in HK-2 Cells under HG ambience. **(A)** Relative protein expression of PTGS2, NF-κB, JNK, and AKT in the HK-2 Cells of the control, HG, and HG + LTD groups by western blot analysis. Representative bands show the relative expression of PTGS2, NF-κB, JNK, and AKT. **(B)** The fluorescence intensity (red) indicated the expression level of P-P65 in HK-2 of the control, HG, and HG + LTD groups by immunofluorescence. Red and blue fluorescence represent P-P65 and the cell nucleus, respectively.

To dissect the effect of high glucose on PTGS2, nuclear factor kappa-light-chain-enhancer of activated B cells (NF-κB), c-Jun N-terminal kinases (JNK), and AKT expression *in vitro*, we exposed cultured HK-2 cells to glucose (30 mmol/L) and showed an upregulation of PTGS2, NF-κB, JNK, and AKT expression (Figure 8A), whereas the expression pattern was reversed by LTD. Moreover, fluorescence microscope data showed that when compared with the control group, an upregulation of P-P65 expression could be seen in the groups treated with HG, this increase was reversed by LTD (Figure 8B).

## Discussion

In our research, we found that LTD may act on key targets such as PTGS2, NF-κB, JNK, and AKT through a variety of chemical components (such as kaempferol, triptolide, nobiletin, and schottenol), and further exert its multi-component, multi-target, and multi-network renal protection by regulating the AGE-RAGE signaling pathway in diabetic complications, IL-17 signaling pathway, insulin resistance, and TNF signaling pathway.

The pathogenesis, pathological process, and related mechanisms of DKD are complex and diverse. Modern medicine mainly treats DKD by controlling blood glucose and preventing or delaying the progressive deterioration of renal function. At present, the therapeutic schemes mainly include angiotensin-converting enzyme inhibitors, angiotensin II receptor blockers, and sodium-glucose cotransporter 2 inhibitors. However, the residual risk of DKD progression and cardiovascular death remains high. In recent years, TCM has demonstrated significant advantages in the treatment of DKD, but the specific mechanism remains unclear. Therefore, it is of great significance to explore the mechanism of TCM in the treatment of DKD.

Studies have found that *Tripterygium wilfordii Hook.F.* and its extracts can effectively reduce proteinuria and protect renal function. Its pharmacological mechanism is related to anti-inflammation, anti-oxidation, anti-fibrosis, and anti-glomerular sclerosis. The mechanism is achieved by balancing Th1/Th2 cells, regulating macrophage infiltration, and regulating p38 mitogen-activated protein kinases (MAPK), nuclear factor kappa-light-chain-enhancer of activated B cells (NF-κB), transforming growth factor-β(TGF-β), Wnt/β catenin signaling, AKT serine/threonine kinase (AKT), and neurogenic locus notch homolog protein 1(Notch1) (Huang et al., 2020). Ma et al. applied tripterygium glycosides combined with irbesartan to explore the effect on urinary podocyte excretion in DKD patients, and the results indicated that the combination of the tripterygium glycosides and irbesartan was effective for preventing podocyte injury in DKD patients, which might be achieved by down-regulating the expressions of connective tissue growth factor (CTGF), osteopontin and TGF-β(Ma et al., 2013). The research by Ge et al. confirmed that *Tripterygium wilfordii Hook.F.* extract could reduce the urinary protein level of DKD patients, and was a new, potentially effective, and safe herb for the treatment of DKD with proteinuria (Ge et al., 2013). Modern studies have unveiled the efficacy of *Trichosanthes kirilowii Maxim* in lowering blood glucose, anti-inflammation, and regulating immunity (Lu et al., 2018; Lu et al., 2019).

To further study and explore the pharmacodynamic basis and potential biological mechanism of LTD in the treatment of DKD, we used network pharmacology and molecular docking to screen 53 compounds of LTD to obtain 126 intersecting targets of diseases and herbs, then construct a protein interaction network, conduct GO function enrichment analysis and KEGG pathway enrichment analysis, then obtained four relevant important components, namely, kaempferol, triptolide, nobiletin, and schottenol, respectively. It was speculated that these extracts were the key components of LTD in the treatment of DKD. Kaempferol has a variety of pharmacological properties, including anti-inflammation, anti-oxidation, and anti-tumor, and it has attracted wide attention as an anti-diabetic herb (Calderón-Montaño et al., 2011). Kaempferol has been found to improve kidney injury and fibrosis by promoting the release of glucagon-like peptide-1 (GLP-1)and insulin and inhibiting RhoA/Rho kinase (Sharma et al., 2020). Triptolide has effects on anti-rheumatism, anti-inflammation, immune regulation, and anti-tumor (Qiu and Kao, 2003; Noel et al., 2019; Huang et al., 2020). *Tripterygium wilfordii Hook.F.* and its extracts are often used in the treatment of a variety of chronic kidney diseases (Chen et al., 2010; Liu et al., 2015; Wang et al., 2017), especially DKD (Li et al., 2019; Liang et al., 2021). It has been demonstrated that nobiletin has anti-inflammatory, anti-oxidant, and anti-cancer properties, and is considered to be a potential therapeutic agent for the prevention of diabetes and diabetic complications. Its role is achieved by regulating the release of inflammatory cytokines, the acetylation of NF-κB, and the expression of Sirtuin 3/6 through the Toll-like receptor/Myeloid differentiation factor 88/NF-κB signaling pathway (TLR/MyD88/NF-κB signaling pathway (Lee et al., 2021). Recent studies have shown that schottenol can regulate mitochondrial activity and affect cell metabolism (Badreddine et al., 2015). In summary, the 4 key core compounds of LTD have anti-inflammatory and anti-oxidant effects in the treatment of DKD, and some of them have been verified by experiments, further confirming the pharmacodynamic material basis of LTD in the treatment of DKD.

According to the analysis of the results of the herb-component-target-pathway network, we found that targets such as PTGS2, RELA, AKT1, and MAPK8 might be the key targets for the treatment of DKD. PTGS2 is a key enzyme that catalyzes the conversion of arachidonic acid to prostaglandins, and it is also an important substance that mediates insulin resistance. PTGS2 can affect renin release and regulation of the renin-angiotensin-aldosterone system (RAAS), as well as renal blood flow and hemodynamics (Nasrallah et al., 2016). RAAS is an important medium for the occurrence and development of DKD (Rahimi, 2016). It has been found that the expression of PTGS2 is increased in KK-Ay mice of different weeks of age, and they show signs of glomerular injury such as glomerular mesangial matrix thickening and nodular sclerosing lesions (Yabuki et al., 2010). DKD is often accompanied by podocyte hypertrophy, shedding, and apoptosis, and podocyte injury is an important factor in the progression of DKD (Zhang et al., 2020). A recent study found that the expression level of PTGS2 was positively correlated with the release of inflammatory cytokines and could mediate the low-density lipoprotein receptor (LDLr) pathway, leading to lipid accumulation and injury of podocytes. At the same time, inhibition of PTGS2 could reduce the injury of podocytes (Ma et al., 2019). Moreover, overexpression of PTGS2 increases the expression of the renin receptor, which would cause local activation of podocytes RAAS, leading to podocyte injury (Cheng et al., 2011). RELA is one member of the NF-κB family, one of the essential transcription factors under intensive study. NF-κB signaling pathway plays an important role in the pathophysiology of DKD. Persistent hyperglycemia and insulin resistance will lead to the increase of advanced glycation end products and excessive production of reactive oxygen species (ROS), leading to NF-κB activation. Excessive activation of NF-κB can lead to apoptosis of vascular endothelial cells. At the same time, NF-κB as an inflammatory mediator can activate a series of inflammatory reactions in the kidney, thereby aggravating kidney damage and accelerating the occurrence and development of DKD (Tóbon-Velasco et al., 2014; Yang et al., 2019; Ke et al., 2021). MAPK8 is a member of the MAPK family. The mammalian MAPK family of kinases includes three subfamilies: Extracellular signal-regulated kinases (ERKs), c-Jun N-terminal kinases (JNKs), p38 mitogen-activated protein kinases (p38s). Previous studies reported that the MAPK signaling pathway played an important role in the occurrence and development of DKD. Many studies have also confirmed that different herbs can effectively improve the occurrence and development of DKD by inhibiting MAPK signaling pathway. The research by Malik et al. indicated that apigenin inhibited oxidative stress and fibrosis by down-regulating the phosphorylation of MAPK, thereby improving the kidney injury caused by DKD. These changes closely resembled the effects of ramipril (Malik et al., 2017). Moreover, MAPK8 plays an essential role in the development of insulin resistance and obesity, whereas MAPK8 knockout mice displayed improved adiposity and insulin sensitivity (Wu H. et al., 2019). AKT is a serine/threonine kinase that regulates adaptation to many cellular stress induction processes. Phosphoinositide 3-kinases (PI3K)/AKT signaling pathway is closely related to glomerular mesangial cell dilatation, podocyte apoptosis, and renal tubular injury. Upregulation of the PI3K/AKT pathway has a protective effect on CKD. PI3K/AKT pathway plays a renal protection role in the DKD model by inhibiting inflammation, apoptosis, and fibrosis, as well as the expression of antioxidants (Mohamed et al., 2022). In agreement with the previous studies stating that PTGS2, NF-κB, JNK, and AKT expression are upregulated in HK-2 cells under HG ambience, and the expression pattern was reversed by LTD under our experimental conditions. In summary, the above targets all play important roles in the occurrence and development of DKD.

Through GO function enrichment analysis, it was found that LTD exerted effects on the treatment of DKD mainly by participating in such biological processes as response to lipopolysaccharide, response to molecule of bacterial origin, response to extracellular stimulus, membrane raft, membrane microdomain, plasma membrane raft, nuclear receptor activity, ligand-activated transcription factor activity, RNA polymerase II-specific DNA-binding transcription factor binding, etc., demonstrating the multifunctional nature of LTD component. The results of the KEGG pathway enrichment analysis showed that it was mainly involved in AGE-RAGE signaling pathway in diabetic complications, IL-17 signaling pathway, insulin resistance, TNF signaling pathway, etc. The AGE-RAGE signaling pathway is closely related to diabetes mellitus and its complications. With the occurrence of insulin resistance, excessive glucose in the blood will cause non-enzymatic glycosylation of proteins, lipids and DNA in the body, resulting in excessive advanced glycosylation end products (AGEs), which are widely distributed in various tissues (Behl et al., 2016). AGEs mainly play a biological role by binding to the receptors of advanced glycation end products (RAGE) on the cell membrane. RAGE is a member of the cell surface immunoglobulin superfamily multi-ligand receptor (Fukami et al., 2014). RAGE in combination with AGEs can activate and promote inflammatory reactions and oxidative stress, leading to other complications of insulin resistance and diabetes mellitus (Ramasamy et al., 2011). Previous studies have found that insulin resistance is a common early change in CKD patients, especially in DKD (Xu and Carrero, 2017), and oxidative stress caused by persistent hyperglycemia is the core mechanism of DKD. Excessive production of ROS under high glucose conditions accelerates mitochondrial DNA damage and mitochondrial function indirectly induces insulin resistance and aggravates renal damage (Wu M. et al., 2019; Carré and Affourtit, 2019). Increased levels of AEGs and excessive production of ROS activate the NF-κB pathway, ultimately leading to apoptosis of vascular endothelial cells. As an inflammatory mediator, NF-κB can also trigger a series of inflammatory reactions in the kidney, thereby aggravating kidney damage and hastening the onset and progression of DKD (Tang et al., 2021). Therefore, it can be speculated that LTD may treat DKD by improving insulin resistance, inflammatory response, oxidative stress, and other mechanisms.

In addition, the core components in the top 4 of the Degree value in the herb-component-target-pathway network diagram of LTD were molecularly docked with the core targets in this study. The results showed that the docking effect of the model is generally good and the structure is stable. The PTGS2 and RELA have relatively low binding energies for docking with most components. These results suggested that LTD might treat DKD by targeting PTGS2 and RELA.

## Conclusion

In summary, the mechanism of LTD in the treatment of DKD was analyzed and discussed in this paper by network pharmacology and verified by molecular docking analysis, western blotting, and immunofluorescence based on the preliminarily screened action targets. The results indicated that the therapeutic efficacy of LTD on DKD might be achieved by decreasing the expression of PTGS2, NF-κB, JNK, and AKT, which might improve insulin resistance, inflammation, and oxidative stress. Moreover, from the perspective of holism in TCM, the multi-component and multi-target synergistic therapeutic effects of TCM are revealed, which is of certain guiding significance for further confirming the key active components of LTD in the treatment of DKD and the development of new herbs in the later stage. We will focus on the study related to the treatment of DKD with LTD to clarify the efficacy and specific mechanism of LTD in intervening DKD.

## Data Availability

The original contributions presented in the study are included in the article/Supplementary Material, further inquiries can be directed to the corresponding authors.
